# Rare clinical insight: esophageal atresia discovered in an adult

**DOI:** 10.11604/pamj.2024.48.72.43959

**Published:** 2024-06-27

**Authors:** Shakuntala Pandey, Bali Thool

**Affiliations:** 1 Department of Obstetrics and Gynaecology, Shrimati Radhikabai Meghe Memorial College of Nursing, Sawangi Meghe, Wardha, India

**Keywords:** Esophageal atresia, adult, dysphagia, esophageal reconstruction

## Image in medicine

The rare congenital disorder known as esophageal atresia (EA), which is defined by an interrupted esophagus, is primarily identified in newborns. Adult appearances are extremely rare and create major difficulties for both diagnosis and treatment. The symptoms of esophageal atresia usually appear in the neonatal stage and include profuse drooling, choking, and difficulties in feeding. Adult occurrences are uncommon and typically manifest as persistent symptoms or are unintentionally found. We describe the case of a 35-year-old woman who had a lengthy history of respiratory infections, dysphagia, and regurgitation when she visited the gastroenterology clinic. Her medical history included sporadic aspiration pneumonia and a persistent cough that had been treated symptomatically. A barium swallow examination performed as part of the first evaluation showed an abrupt end of the esophagus, indicating the possibility of a congenital abnormality. Due to the patient's chronic complaints and the intricacy of the situation, a multidisciplinary team of anesthesiologists, thoracic surgeons, and gastroenterologists was gathered. An esophagus reconstruction procedure was scheduled as a surgical repair. The patient underwent excellent surgical tolerance and underwent postoperative monitoring in the intensive care unit. Over several weeks, she progressively transitioned from nasogastric tube feeding to oral consumption. Postoperative complications included transient anastomotic leak, managed conservatively with antibiotics and drainage.

**Figure 1:**
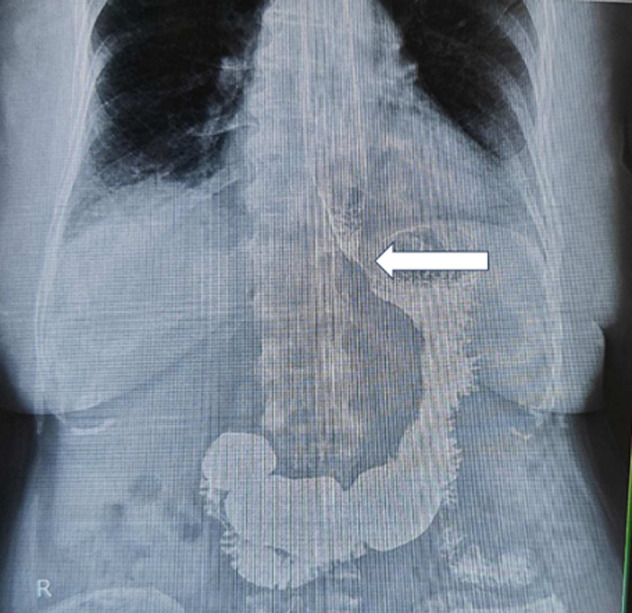
barium meal X-ray of esophageal atresia

